# Total Delay in Treatment among Smear Positive Pulmonary Tuberculosis Patients in Five Primary Health Centers, Southern Ethiopia: A Cross Sectional Study

**DOI:** 10.1371/journal.pone.0102884

**Published:** 2014-07-21

**Authors:** Anteneh Asefa, Wondu Teshome

**Affiliations:** School of Public and Environmental Health, College of Medicine and Health Sciences, Hawassa University, Hawassa, Ethiopia; McGill University, Canada

## Abstract

**Introduction:**

The global burden of Tuberculosis (TB) remains enormous. Delay in TB diagnosis may lead to a higher infectious pool in the community and a more advanced disease state at presentation increasing the risk of mortality. This study is conducted to determine the total delay before treatment among smear positive Pulmonary Tuberculosis (PTB) patients.

**Methods:**

A health institution based cross sectional study was conducted in five primary health centers in southern Ethiopia from June to December 2012. A total of 328 smear positive PTB patients were enrolled in the study. A structured and pre-tested questionnaire was used. Median patient, diagnostic, and treatment delays were calculated to determine the total delay. Multiple logistic regression analysis was used to identify factors associated with total delay.

**Results:**

The median patient, diagnostic, treatment and total delays measured in days were 30 (IQR 20.2, 60), 7 (IQR: 3, 14), 3 (IQR: 1, 4) and 45 (IQR: 34.5, 69.5) days respectively. Patients for whom treatment was not initiated within 45 days of onset of symptom(s) (total delay) constituted 49% of the study participants (59.5% among males and 39.2% among females; P<0.001). Total delay was found to be associated with: being female [AOR  = 0.34, 95% CI: 0.18–0.62], having attended tertiary level education [AOR  = 0.11, 95% CI: 0.02–0.55], perceived severity of stigma during the current TB disease course [AOR = 2. 18, 95% CI: 1.07, 4.42] and living in houses with higher family size [AOR = 0.26, 95% CI: 0.11, 0.61].

**Conclusion:**

Total delay in treatment of TB is still high in the study area. Patient's sex, perceived stigma, educational status and family size are significantly contributing for total delay. Therefore, a concerted effort should be taken in order to improve health seeking behavior of the community on TB and to reduce delays from seeking care after experiencing TB symptoms.

## Introduction

Since 1993, when the World Health Organization (WHO) declared tuberculosis (TB) a global public health emergency due to the steady increase of the disease worldwide, TB remained a major global health problem. New cases of TB have been falling for several years and fell at a rate of 2% between 2011 and 2012. The TB mortality rate has decreased by 45% since 1990 and the world is on track to achieve the global target of a 50% reduction by 2015. The best estimate of the Case Detection Rate (CDR) for all forms of TB globally in 2012 was 66% (range, 64–69%), up from 53–59% in 2005 and 38–43% in 1995. Despite those achievements, the global burden of TB remains enormous. In 2012, there were an estimated 8.6 million new cases of TB (13% co-infected with HIV) and 1.3 million deaths from TB [1].

In 1995 the DOTS (Directly Observed Treatment, Short course) strategy was established as the key intervention to achieve TB control worldwide. Between 1995 (when reliable records began) and 2006, a total of 31.8 million new and relapse cases, and 15.5 million new smear-positive cases were notified by DOTS programmes [2]. Later on, in 2006, WHO launched the stop TB strategy as an approach to TB care and control [3].

Ethiopia is one among the 27 high Multi Drug Resistant Tuberculosis (MDR-TB) burden countries and the rate of TB treatment success in the country was 83% in 2011 [1].

Early diagnosis, prompt, and effective therapy are the key elements of the TB control programme. Delay in diagnosis results in increased infectivity in the community and it is estimated that an untreated smear-positive patient can infect, on average, 10 contacts annually and over 20 during the natural history of the disease until death [3]–[5]. Delay in TB diagnosis may also lead to a more advanced disease state at presentation, which contributes to late sequelae and overall mortality. Smear-positive cases are more likely to infect other individuals [1]–[4].

Various studies in Ethiopia revealed that diagnostic and treatment delays in TB are still prevailing problems which contribute to the high TB burden in the country [6]–[13].

Treatment delay among PTB patients may be due to patients' delay in seeking health care, health care providers' delay in making prompt and accurate diagnosis with subsequent initiation of treatment, a worse prognosis owing to the presence of extensive disease and poor clinical condition or any combinations of these [13]. Furthermore, rural residence, low access (geographical or socio-psychological barriers), uncertainty about where to go for care, initial visit to traditional healer, old age, low educational level, low awareness of TB, incomprehensive beliefs, self-treatment, and perceived severity of stigma were also reported to be associated with diagnostic delays [4], [14].

However, studies suggest that the reported high treatment success rate of DOTS may be supplemented by measures to shorten the delay in diagnosis, which may result in reduction of infectious cases and better TB control [13], [15].

Smear positive PTB patients are known to contribute the highest infectious pool when compared with other types of TB. Many other studies conducted in this area have considered all types of TB with a further breakdown during analysis and it may not be representative for smear positive PTB patients. Thus, this study is aimed at assessing the extent of total delay in TB treatment among smear positive PTB patients and factors contributing to the delay.

## Materials and Methods

### Study area

The study was conducted in six primary health centers namely: Kuyera, Yirgalem, Wosha, Mesenkela, Chuko and Kella Health Centers. The health centers are located in Southern Ethiopia within a radius of 60 kms from Hawassa, the capital city of Southern Nations Nationalities and People's Region. In 2012/2013, there were 19,140 PTB patients in the region from which 11,779 patients were smear positive TB patients [16].

### Study design

A health institution based cross sectional mono-strand design which employed quantitative data collection techniques was conducted to assess the total treatment delay among smear positive PTB patients in the study health facilities. The study was conducted between June 2012 and December 2012.

### Study population and sampling

The study subjects of this study were smear positive PTB patients who were following their DOTS treatment in the study facilities. The sample size for the study was determined using the formula to calculate sample sizes for a single population proportion under the following assumptions: confidence interval of 95%, a proportion of 59% for total delay more than 30 days among smear positive patients [8], margin of error of 5%, and an expected non response rate of 10%. Hence the calculated sample size was 371. But, since the size of the target population (number of smear positive PTB patients in the study area) in 2011/2012 was small (1182), we have considered a finite population correction for sample size. This made the final sample size of the study 311. However, 328 smear positive PTB patients were included in this study, which makes the response rate of the study 105%. Samples were proportionately allocated to the six health facilities based on their estimated annual flow of smear positive PTB patients. Then after, newly diagnosed smear positive PTB patients were selected consecutively from the aforementioned health facilities within the specified time period until the required sample size was met.

### Study variables

- Dependent variable: Duration in days of delay in commencing anti-TB treatment, a binary outcome of weather patients were delayed or not to initiate treatment (presence/absence of total delay).
- Independent variables: Sex, educational status, age, family size, occupation, residence, income, distance to health facility, attitude towards traditional healers, attitude towards spiritual healing, severity of disease, perceptions towards TB and its treatment, knowledge of TB disease.

### Operational definition of terms

Smear positive PTB patients: patients with two or more sputum smears which are positive for acid fast bacilli.

Total treatment delay: the time interval between dates of onset of the main TB symptom as reported by patients as their chief complaints, and initiation of anti TB treatment, in this case more than 45 days (that is the median total delay).

Stigma scores: patients were asked to rate (using scale of 3) their responses to the following four questions: feeling ashamed, feeling of hiding the disease, feeling that TB affects relation with others and preference of feeling isolated. The responses were coded as 1 =  strongly agree, 2 =  neutral and 3 =  strongly disagree. The scales were added up for all patients and those with a score of 11/12 were coded as no/mild stigma, and those with score ≤7 were recorded as severe stigma and the remaining values as moderate stigma score.

### Data collection and analysis

The data were collected using structured and pretested questionnaire by nurses/health officers working in TB clinics of the selected health facilities. Data collectors have received an on-site training on the tools prior to data collection.

**Figure 1:**
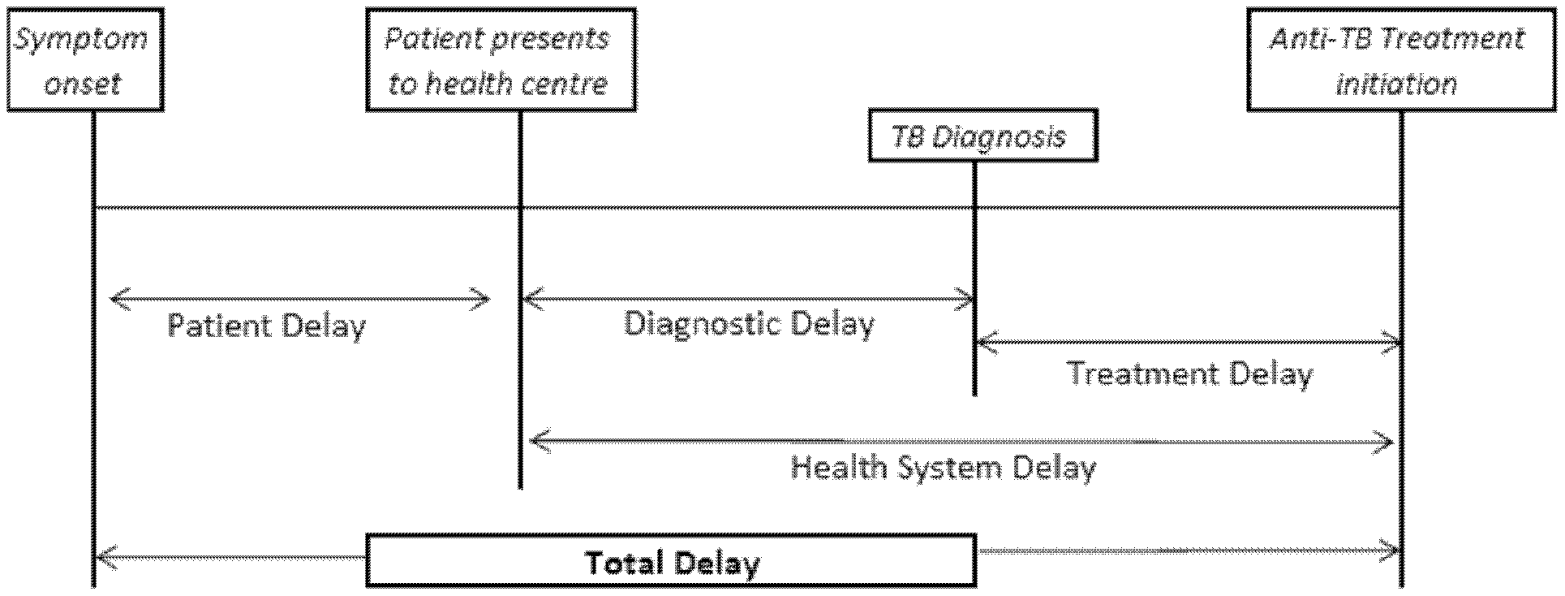
Different categories of delay and their contribution to total delay.

The data were entered, cleaned and analyzed using SPSS version 16.0. Treatment delays were calculated using the scheme displayed in figure 1.

Patients for whom anti-TB treatment was not initiated within the first 45 days of onset of symptoms were considered to experience total delay. Factors that contribute to delay in treatment were determined using multivariate logistic regression. All statistical tests were carried out assuming alpha of 0.05. Mean (median) patient, diagnostic and treatment delays were calculated in addition to identification of total delay. Chi-square and Mann-Whitney tests were used to identify whether there is a significant difference of total delay between selected categorical variables.

### Ethical approval

This study was conducted after an ethical clearance was secured from the Institutional Review Board of the College of Medicine and Health Sciences, Hawassa University. Permission letters to conduct the study was also obtained from the health facilities included in the study. Moreover, verbal consent was obtained from all study participants who participated in the study. Written consent was not obtained from study participants as verbal consent meets the criteria for ethical approval from the institutional review board and the study used only interview as data collection method. Verbal consents were sought after explaining benefits of the study and assurance of confidentiality to study participants; participants' consent was documented using check boxes which are put on every questionnaire's information sheet.

## Results

### Basic profiles of study participants

A total of 328 smear positive PTB patients were included in the study. The median age was 28 years (IQR: 20–30 years). Most study participants (56.7%) were in the age range between 21 and 30 years and little more than half (51.8%) were females. More than half of the study participants (65.9%) had income less than or equal to 550 Birr per month and around 4 out of 5 study participants had not attended formal education at all or were only able to read and write (Table 1).

**Table 1:** Socio-demographic and other baseline characteristics of study participants, southern Ethiopia, June-December 2012.

Variable	Frequency (%)
**Age in completed years**	
15–20	84 (25.6)
21–30	186 (56.7)
> = 31	58 (17.7)
**Total**	**328 (100.0)**
**Median (IQR)**	28 (20, 30)
**Sex**	
Male	158 (48.2)
Female	170 (51.8)
**Total**	**328 (100.0)**
**Residence (patients' response)**	
Urban	160 (48.8)
Rural	168 (51.2)
**Total**	**328 (100.0)**
**Time taken to reach health center (self report) (n = 326)**	
≤30 minutes	178 (54.6)
31–60 minutes	102 (31.3)
>60 minutes	46 (14.0)
**Total**	**326 (100.0)**
Estimated Income, Birr (n = 282)*	
≤550/month	216 (65.9)
>550/month	66 (20.1)
**Total**	**282 (100.0)**
**Educational status**	
No formal education/read and write only	276 (84.2)
Elementary/high school	26 (7.9)
Tertiary	26 (7.9)
**Total**	**328 (100.0)**
**Crowdedness measures**	
1–2 persons/room	148 (45.1)
2.1–3.5 persons/room	100 (30.5)
> = 3.6 persons/room	80 (24.4)
**Total**	**328 (100.0)**

* On average 1 USD was around 17.5 birr during the data collection period.

Among the study participants, 178 (54.6%) reported to have taken less than 30 minutes to arrive at their respective health facility during the date of interview. The number of persons per room in the respondents' residential home was more than 3.5 persons in nearly one-fourth (24.4%) of the study participants (Table 1).

### Major complaints of outpatient department (OPD) visits by patients

**Figure 2:**
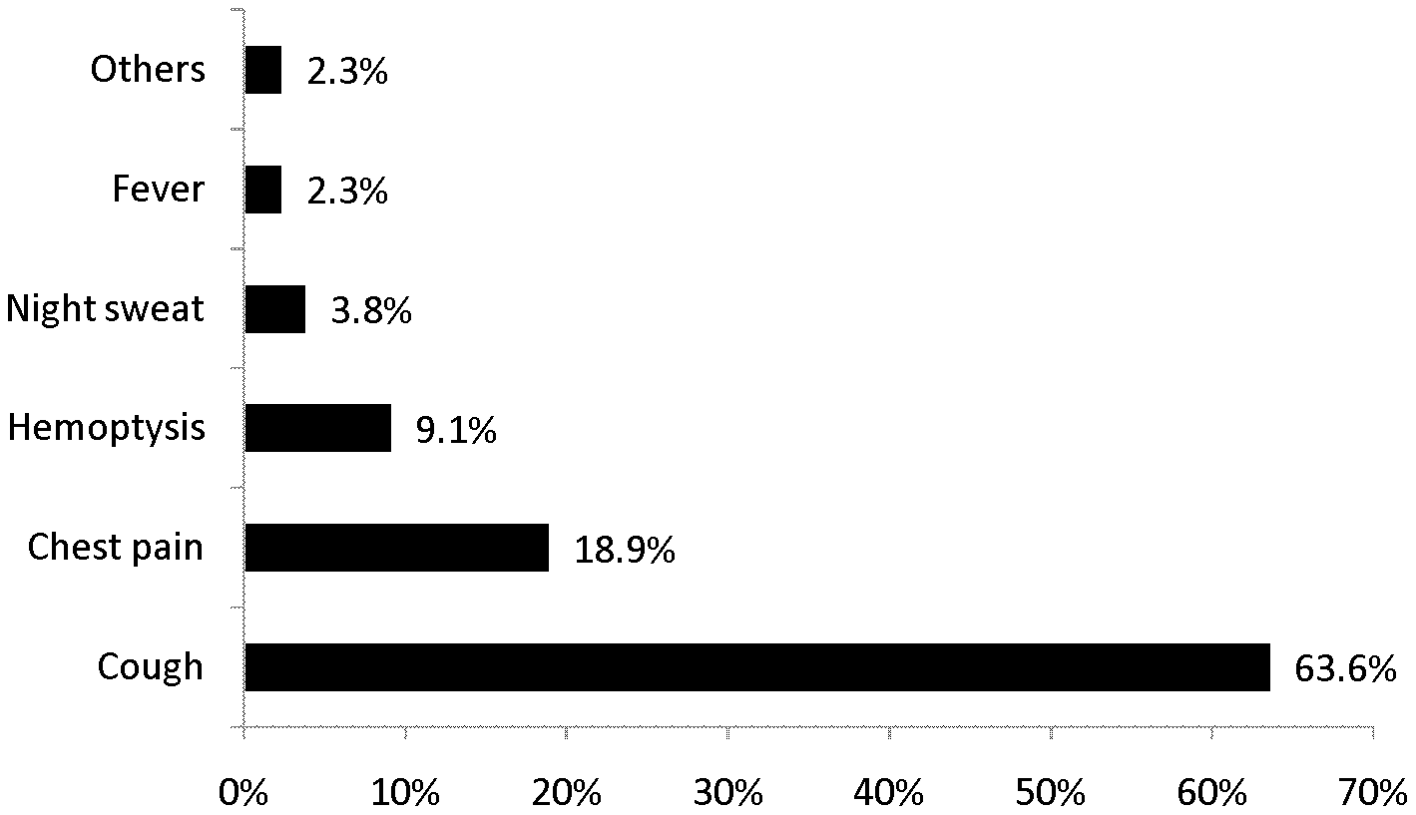
Complaints which made patients seek medical care, Southern Ethiopia, June-December 2102.

Cough was the most important symptom that derived patients to seek medical care from the health centers. More than 63% of patients had complaints of cough as a major symptom triggering to seek medical care. This was followed by chest pain accounting for around 19% of the reason for OPD visits (Figure 2).

### First visit for care and duration of symptoms before visiting the current health institution

Relatively large proportion of patients (46.3%) first visited the current health institution for medical care for their major complaint. The remaining patients had visited private clinic (35.4%), had applied self treatment at home hoping that symptoms would go away soon (14.6%), visited traditional healers (1.2%) or sought religious healing (2.4%).

**Figure 3:**
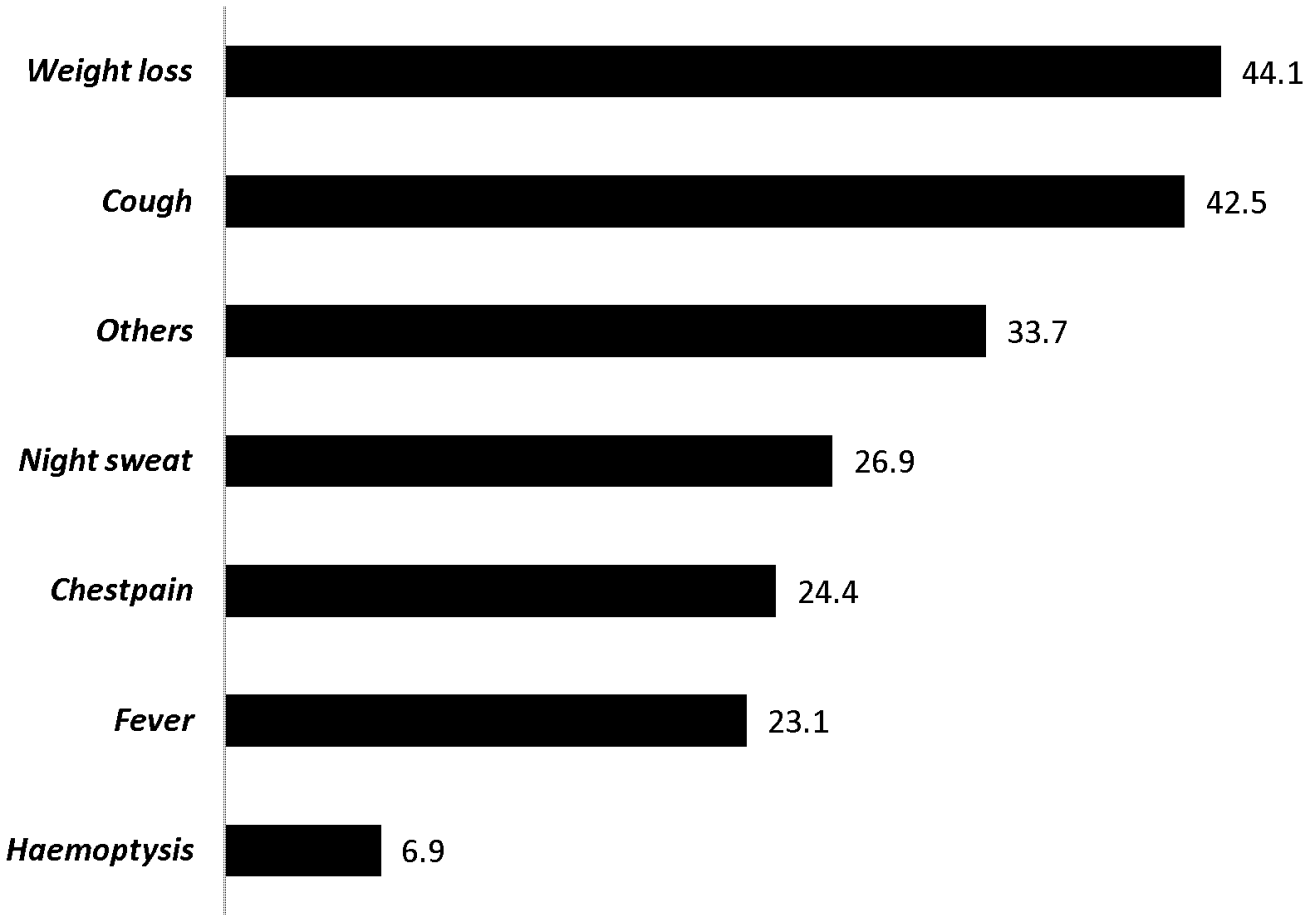
Mean duration of symptoms in days among PTB patients, Southern Ethiopia, June-December 2012.

Weight loss is the symptom with higher mean duration before seeking treatment where as haemoptysis had the shortest mean duration, 44.1 (SE of mean = 12.0) vs. 6.9 (SE of mean  = 1.2) days respectively. Patients with cough presented to the current health facility after a mean duration of 42.5 (95% CI: 36.4, 48.6) days (Figure 3).

### Knowledge and stigma score

Two hundred seventy two patients (82.6%) have ever heard of TB before their current TB disease. No any gender difference was observed for ever hearing about TB (p>0.05). Mass media (45.7%) and TB disease among friends (45.7%) were the relatively commonest sources of information for TB; followed by health workers, other friends/close relatives and school education acting as a source of information for 39.0%, 32.9% and 17.7% of respondents respectively.

Patients were asked three main questions to assess the correctness of the information they had. In general the knowledge level can be judged as ‘limited’ with 59.1%, 53.7% and 75.6% knowing that TB is not hereditary, TB is contagious and TB is curable respectively.

Further gender segregation of the data indicated that, in terms of the contagiousness and hereditary perceptions of TB, no significant difference was observed among males and females (p>0.05 for both variables). However, the fact that TB is a curable disease is more reported from males than females (OR = 3.67; 95% CI: 1.38, 9.77).

Overall the stigma related responses are not significantly different for males and females (p>0.05 for all the questions). A total of 112 (34.1%) patients felt ashamed of their current TB disease (32.9% among males and 35.3% among females). Moreover, 68 (20.7%) patients had the feeling that they had to hide their TB disease from others (16.5% among males and 24.7% among females) (Table 2).

**Table 2:** Stigma indicating questions stratified by sex of study participants, southern Ethiopia, June-December 2012.

S. No	Stigma related questions	Scale	Sex		P-value
			Male	Female	
1	**Do you feel ashamed of your TB disease (n = 328)**	Strongly agree	52 (32.9)	60 (35.3)	p>0.05
		Average	28 (17.7)	26 (15.3)	
		Do not agree at all	78 (49.4)	84 (49.4)	
2	**Do you have to hide others that you have TB disease (n = 328)**	Strongly agree	26 (16.5)	42 (24.7)	p>0.05
		Average	38 (24.1)	34 (20.0)	
		Do not agree at all	94 (59.5)	94 (55.3)	
3	**Does TB affect your relation with others (n = 328)**	Strongly agree	34 (21.5)	42 (24.7)	p>0.05
		Average	40 (25.3)	30 (17.7)	
		Do not agree at all	84 (53.2)	98 (57.7)	
4	**Do you prefer to live isolated due to your current illness (n = 322)**	Strongly agree	66 (42.9)	62 (36.9)	p>0.05
		Average	16 (10.4)	38 (22.6)	
		Do not agree at all	72 (46.8)	68 (40.5)	
5	**Are girls unable to decide to seek TB care on their own (n = 324)**	Strongly agree	10 (6.4)	10 (5.9)	p>0.05
		Average	30 (19.2)	40 (23.8)	
		Do not agree at all	116 (74.4)	118 (70.2)	

### Patient, diagnostic and Treatment delays

The delays in days are displayed in Table 3. It was found that the median patient, diagnostic, treatment and total delays measured in days were 30 (IQR 20.2, 60), 7(IQR: 3, 14), 3 (IQR: 1, 4) and 45 (IQR: 34.5, 69.5) days respectively. The median health systems delay was 11 (IQR: 7, 18) days. Patient and diagnostic delays were not significantly different across sex of study participants. However, there was a higher total delay among males (p = 0.004), and marginally lower treatment delay among females (p = 0.015).

**Table 3:** Different categories of total delay segregated by sex of study participants, southern Ethiopia, June-December 2012.

Delays	Total (for both males and females)	Male	Female	P-value (Mann-Whitney test)
**Patient delay (days)**				
Mean (±1 SD)	42.6 (39.5)	42.5 (28.6)	42.6 (47.9)	
Median	30	30	30	0.347
IQR	(20.3, 60)	(20, 60)	(25.0, 31.3)	
**Diagnostic delay(days)**				
Mean (±1 SD)	13.5 (17.3)	16.4 (21.9)	10.9 (11.1)	
Median	7	7	7	0.068
IQR	(3, 14)	(3, 14)	(3, 14)	
**Treatment delay (days)**				
Mean (±1 SD)	3.7 (5.1)	3.1 (4.2)	4.2 (5.9)	
Median	3	2	3	0.015*
IQR	(1, 4)	(1, 4)	(2, 4)	
**Health systems delay (days)**				
Mean (±1 SD)	17.3 (18.1)	19.9 (22.2)	15.0 (12.9)	
Median	11	11	9	0.113
IQR	(7, 18)	(8, 17.0)	(6.18.0)	
				
**Total Delays (days)**				
Mean (±1 SD)	59.8 (44.2)	61.8 (33.4)	57.9 (52.4)	
Median	45	53	41	0.004*
IQR	(34.8, 69.3)	(36, 78)	(34.0, 65.0)	

* Significantly associated.

Among the TB patients for whom the total treatment delay was calculated (n = 306), 150 (49%) have experienced total treatment delay. When stratified by sex, 59.2% males and 39.5% females had a total TB treatment delay (P<0.001). In this study, total delay was associated with being female, which revealed that females are less likely to experience total delay than males [AOR  = 0.34, 95% CI: 0.18–0.62]. Similarly, patients with tertiary level of education were less likely to experience total delay in treatment than those with primary or secondary level of education [AOR  = 0.11, 95% CI: 0.02–0.55]. Furthermore, patients who live in dense family were less likely to have a total delay in initiating treatment than their counterparts [AOR  = 0.26, 95% CI: 0.11–0.61] (Table 4).

**Table 4:** Multivariate analysis of risk factors of total delay among smear positive PTB patients, southern Ethiopia, June-December 2012.

Variables	Total Delay		Crude OR (95% CI)*	Adjusted OR (95% CI)* †
	Yes	No		
**Sex**				
Male	88	60	1.00	
Female	62	96	0.44 (0.28, 0.70)**	0.34 (0.18, 0.62)**
**Residence**				
Urban	70	76	1.00	
Rural	80	80	1.09 (0.69, 1.70)	1.06 (0.48, 2.31)
**Age in completed years**				
≤20	40	40	1.00	
21–30	86	88	0.98 (0.58, 1.66)	1.48 (0.70, 3.13)
≥31	24	28	0.86 (0.43, 1.73)	0.79 (0.31, 2.00)
**Educational status**				
Primary/secondary	18	8	1.00	
Tertiary	4	22	0.8 (0.02, 0.31)**	0.11 (0.02, 0.55)**
No formal education/able to read and write	126	126	0.45 (0.19, 1.08)	0.78 (0.27, 2.26)
**Estimated monthly income (In birr)**				
≤500	104	92	1.00	
>500	26	40	0.58 (0.33, 1.01)	0.60 (0.27, 1.36)
**Family density**				
<2 persons per room	76	60	1.00	
2.1–3.5 persons per room	44	48	0.72 (0.43, 1.23)	0.40 (0.20, 0.82)*
≥3.6 persons per room	30	48	0.49 (0.28, 0.87)**	0.26 (0.11, 0.61)**
**Time travelled to arrive at health center**				
≤30 minutes	82	84	1.00	
31–60 minutes	38	58	0.67 (0.40, 1.12)	0.75 (0.35, 1.57)
>60 minutes	30	12	2.56 (1.23, 5.34)*	1.58 (0.59, 4.29)
**First visited facility**				
Nom-medical^‡^	26	30	1.00	
Private clinic	52	60	1.00 (0.53, 1.90)	1.41 (0.65, 3.06)
Current health center	68	66	1.19 (0.64, 2.22)	1.32 (0.59, 2.93)
**Stigma scores**				
Mild/no stigma	38	46	1.00	
Moderate stigma	72	56	1.56 (0.90, 2.71)	2.18 (1.07, 4.42)**
Severe stigma	36	52	0.84 (0.46, 1.53)	0.78 (0.36, 3.06)

† All variables were controlled for each other, ^‡^Self treatment/traditional healer/religious healer.

*p-value <0.05, **statistically significant.

## Discussion

This study depicts that total delay in treatment among smear positive PTB patients was high (49%). From the total delay, majority of the delay is attributable to patients' delay followed by diagnostic delay. This necessitates the implementation of a cost effective strategy to mitigate those delays in order to hasten the treatment success and reduce transmission of the disease.

In the current study, 81.7% of patients visited health workers for their main symptom which is less than another study conducted in Afar region, Ethiopia (88.4%) and higher than a study conducted in Amhara region, Ethiopia [9], [10]. This may be due to difference in study subjects of the Afar's study which included patients with all forms of TB. But, a study which included PTB patients from ten districts of Tigray region, Ethiopia, revealed that 54% of patients first received care from formal health providers which is far less than the finding of this study [11]. This difference might be as a result of the fact that there is a diverse cultural and religious belief in Ethiopia across regions, and this difference is believed to contribute to variation in health seeking behavior [6], [9], [10], [14], [17].

Relatively very small proportion (1.2%) of study participants reported to have first visited traditional healers for their major complaint in this study as compared to other similar study conducted in Uganda (9.1%) [18]. Visiting traditional healers for the first time after onset of symptom(s) was also reported in other Ethiopian studies in Tigray (13%), East Wollega (4.1%), and Amhara (27%) [7], [11], [12]. Health cadres should try to involve traditional healers in the provision of care for TB patients, which was found to be productive in Hlabisa district, South Africa [19]. A similar recommendation was also forwarded from a research conducted in Malawi which suggested involvement of traditional healers to detect and refer TB patients to health facilities [20].

A mean duration of 42.5 days was experienced by patients with cough before appearing at their respective health center. This figure is much higher than the recommended date of visit (14 days) which is forwarded by the World Health Organization [21]. In this study it was observed that patients visit health facilities very quickly when they experience worrying symptoms (e.g. haemoptysis). This may indicate that patients are reluctant for less worrying/gradually progressing symptoms like weight loss or cough.

There is general understanding that considerable proportions of TB patients in developing countries don't have adequate knowledge on TB. For example, 4.7% of PTB patients don't know that TB is contagious in a study carried out in Vietnam [22] which is far less than the finding from this study (53.7%). But, in similar study conducted in Zambia, 57% of respondents reported as TB is a contagious disease [23]. Among the total respondents, 75.6% knew that TB is curable. But there was high proportion of knowledgeable patients in a hospital based study conducted in Thailand (96%) [24] which may be due to the set up difference. Apart from this, 42.9% of men and 36.9% of women strongly agreed with the idea that they want to live isolated (self discrimination) which may be due to fear of transmitting TB, and avoiding gossip and potential discrimination [25]. A study from Dharan, India also depicted as stigma was found to be associated with treatment outcome and delay in seeking care [26] which is in line with the finding of this study.

Female participants of this study were found to be less likely to experience total delay than their counterparts [AOR = 0.34, 95%CI: 0.18–0.62]. This association was also reported by a study conducted in Uganda among smear positive TB patients which showed that males were more likely to experience delay in treatment [AOR = 1.83, 95%CI: 1.02–3.29] [18]. However other studies conducted in Ethiopia (Amhara [7], Afar [9], and Tigray [11] regions), Nigeria [27], Spain [28], and Brazil [29] did not show any difference with regards to gender of participants. This gender difference showed as women seek care faster than men and this is justified by other studies [30], [31].

Educational status of patients was associated with total delay in this study. Several studies conducted also found out that patients with improved educational status were less likely to experience total delay in TB treatment [32], [33]. This could be due to the increased awareness of the TB disease and reinforced by good treatment seeking behavior among educated people. The time taken to arrive at health facility has lost statistical significance when controlled for other variables, however, it is a commonly reported cause of delay in previous studies [32], [33]. This might have happened due to the few number of cases in the >60 minutes group, this is expected as health centers are closer to the community as opposed to hospitals.

Over crowdedness is one of the key risk factors for transmission of TB especially from smear positive patients. The fact that patients living in large families are less likely to delay from care is a positive social effect on treatment seeking for TB, otherwise the transmission of the disease could even be worse in such families.

Finally, this study is not free from measurement errors. The study included only smear positive PTB patients and hence the findings cannot be generalized to smear negative and other types of PTB patients. Furthermore, recall bias is inherent in this study as the total delay was calculated based on the patients' self reported duration before the important time frames to calculate the delays.

## Conclusions

Total delay in treatment of TB is still high in the study area. Patient's gender, literacy level, family size, and perceived personalized stigma contributed their own share for total delay. In addition to this, knowledge of patients on TB is still inadequate and needs further attention. In order to mitigate the consequences of TB, concerned stakeholders need to design successful mechanisms to improve awareness of the community on TB and to reduce delay from seeking care after experiencing TB symptoms.
